# All Types of Age-related Macular Degeneration in One Patient

**DOI:** 10.4274/tjo.14602

**Published:** 2017-12-25

**Authors:** Zafer Cebeci, Nur Kır

**Affiliations:** 1 İstanbul University İstanbul Faculty of Medicine, Department of Ophthalmology, İstanbul, Turkey

**Keywords:** Age-related macular degeneration, retinal angiomatous proliferation, polypoidal choroidal vasculopathy

## Abstract

Herein, we describe a neovascular age-related macular degeneration patient with retinal angiomatous proliferation (RAP) and polypoidal choroidal vasculopathy (PCV) coexisting in the same eye at the time of diagnosis. A 55-year-old woman presented with a history of decreased vision in her left eye. Fundoscopy, fluorescein and indocyanine green angiography, and optical coherence tomography imaging revealed RAP and PCV lesions in her left eye at first diagnosis. The patient received intravitreal ranibizumab therapy but developed tachyphylaxis after the first dose despite having three monthly doses. Switching to intravitreal aflibercept injection in our case resulted in anatomic and functional improvement.

## INTRODUCTION

Neovascular age-related macular degeneration (nAMD), also known as “wet” or “exudative” AMD, is characterized by the abnormal formation of new choroidal vessels with growth under the retinal pigment epithelium (RPE) or in subretinal spaces, resulting in severe vision loss.^1^ Polypoidal choroidal vasculopathy (PCV) features clinically distinguishable orange-reddish lesions beneath the RPE which are caused by dilation of abnormal choroidal vessels. PCV was first reported by Yannuzzi et al.^2^ in 1990, yet there is still debate about whether PCV should be considered a subtype of nAMD or if they are completely distinct entities. Retinal angiomatous proliferation (RAP), a subtype of nAMD, is a pathology in which the vasogenic process of neovascularization starts from the retina to form choroidal neovascularization (CNV) and is strongly associated with soft drusen or reticular pseudodrusen at the macula.^3^ RAP tends to show bilateral involvement and is more common in older patients.^3^ The coexistence of PCV and typical nAMD has been reported in the literature, and although the combination of type 1 and type 3 AMD was also reported, the authors did not provide a detailed description of this case.^4,5,6,7,8^

In this report, we describe a case of nAMD co-presenting with different types of lesions in a patient who responded to aflibercept treatment after developing tachyphylaxis to ranibizumab.

## CASE REPORT

**Figure 1:**
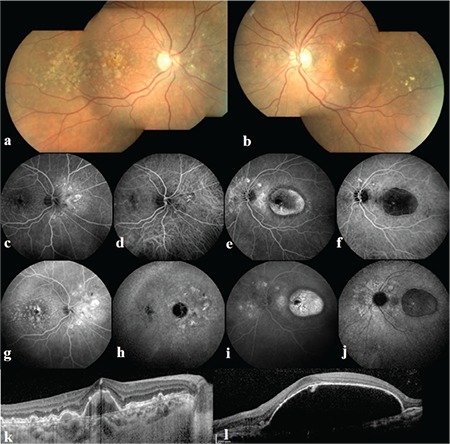
Fundus photography of the right (a) and left (b) eye showing drusen and peripapillary orange-red lesions bilaterally and serous pigment epithelial detachment (PED) on the left eye. Early and late fluorescein angiography and indocyanine green angiography (ICGA) images of the right (c, d, g, h) and left (e, f, i, j) eye. ICGA shows peripapillary hyperfluorescent polypoidal lesions bilaterally and hyperfluorescent spot in the center of hypofluorescent PED on the left eye suggesting a retinal angiomatous proliferation lesion. Spectral domain optical coherence tomography scan of the right macula (k) illustrating drusen and drusenoid PED and serous PED, with subretinal fluid and hyperreflective lesion under the pigment epithelium on the left macula (l)

A 55-year-old white female presented to our clinic with a chief complaint of gradually decreasing vision in her left eye that she had first noticed one month earlier. She had an unremarkable past ocular and systemic history. In her family history, her parents had a diagnosis of AMD but they did not receive any treatment for this pathology. Her best corrected visual acuity was 20/25 in the right and 20/32 in the left eye. Anterior segments were normal bilaterally. Fundoscopic evaluation showed soft drusen on the macula and peripapillary reddish-orange lesions bilaterally. There was also drusenoid retinal pigment epithelial detachment (PED) in the right and serous PED in the left eye (Figure 1a, b). Fluorescein angiography (FA) revealed peripapillary hyperfluorescence in both eyes which increased in late phases and hyperfluorescence in late phases due to serous PED in the left macula (Figure 1c, e, g, i). Indocyanine green angiography (ICGA) showed peripapillary polypoidal hyperfluorescent lesions bilaterally and hyperfluorescent hot-spot in the centre of hypofluorescent PED, suggesting RAP in the left eye (Figure 1d, f, h, j). Spectral domain optical coherence tomography scan of the macula demonstrated drusen and drusenoid PED in the right eye and serous PED with hyperreflective lesion under the RPE and concomitant subretinal fluid in the left eye (Figure 1k, l). Based on examination and imaging findings, we diagnosed the patient with bilateral AMD consisting of different lesion types.

**Figure 2:**
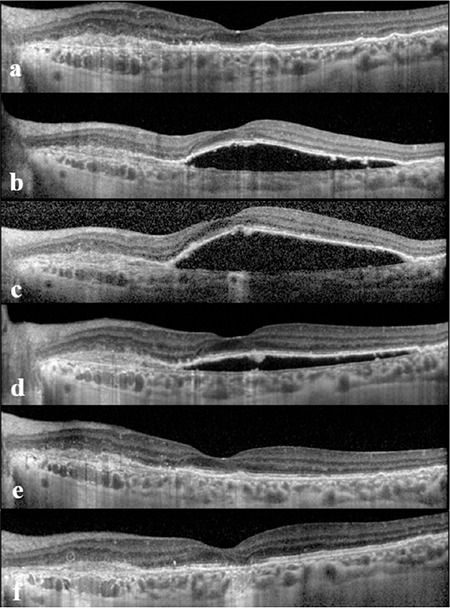
Spectral domain optical coherence tomography images of the left eye one month after first (a), second (b), and third (c) intravitreal ranibizumab injections. Switching to aflibercept, one month after first (d), second (e) injections and after 9 months (f)

Intravitreal ranibizumab (0.5 mg/0.05 mL) injection with three monthly loading doses was planned for the left eye after diagnosis. One month after the first dose, serous PED had totally regressed (Figure 2a), but reappeared after the second dose (Figure 2b) and increased despite a third dose (Figure 2c). One month after the third dose, we switched treatment from ranibizumab to aflibercept (2 mg/0.05 mL). Serous PED decreased one month after the first aflibercept injection and totally resolved after the second injection (Figure 2d, e). The patient received three monthly loading doses and continued with pro re nata protocol. She received a total of five injections during the nine months follow-up after starting to use aflibercept. At the final examination, her vision was 20/25 in the left eye and OCT showed no PED or intra- or subretinal fluid. The PCV lesions on ICGA had totally resolved but a small area of subfoveal atrophy developed during the follow-up period (Figure 2f).

## DISCUSSION

Combinations of PCV and typical nAMD lesions in the same eye or one in each eye of the same patient have been reported in the literature.^4,5,6,7,8^ However, the coexistence of PCV and RAP at the time of diagnosis has not been previously described. In a group of newly diagnosed 155 nAMD patients, Liu et al.^4^ found 3.2% of the cases had mixed lesions, all of them with PCV and typical CNV in the same eye. The authors considered this mixed presentation a third subtype of nAMD. In a series of 289 Japanese patients with PCV, RAP, and typical AMD, Maruko et al.^5^ found that 5.5% of the patients had combined lesions, all with PCV in one eye and typical AMD in the other eye. However, no combination of RAP and PCV was detected in these cases. Pereira et al.^6^ reported that 5.3% of their Brazilian nAMD patients had combined lesions with different types of each in one eye, but the combination of RAP and PCV in the same eye was not reported. In a study assessing the newly diagnosed subtypes of nAMD according to FA alone and FA + OCT images, the authors divided subtypes as type 1 (subRPE), type 2 (subretinal), type 3 (intraretinal), and mixed.^7^ PCV was considered type 1 and RAP as type 3. Using FA + OCT, mixed lesions were detected in 16.9% of 266 eyes and 15.5% of mixed lesions were a combination of type 1 and 3. However, they did not provide further details about the coexistence of PCV and RAP in the same patient or eye. One report included an 86-year-old female patient with unilateral RAP who developed PCV in the fellow eye three years after the initial diagnosis.^8^ Our patient had RAP and PCV in the same eye at the time of diagnosis and she may have presented in a early phase, enabling us to identify the RAP lesion. If the patient presented us later, progression towards the advanced stages might occured and we could have diagnosed as CNV instead of RAP.

Another issue that must be emphasized in our case is the development of tachyphylaxis. Binder^9^ differentiated tolerance from tachyphylaxis and pointed out that tachyphylaxis could occur in a short time when drugs were used repeatedly. There are several potential mechanisms for development of tachyphylaxis in nAMD, including the development of antibodies against anti-VEGF, change in lesion type or neovascular membrane structure, and other pathways of action used by anti-VEGF drugs.^9^ Another possible explanation could be upregulation of pro-angiogenic factors other than VEGF-A.^9^

The combination of PPV, ILM peeling, MB, and gas tamponade may be effective in patients with high myopia and MHRD. However, although the anatomical success is high with this procedure, functional success may be limited due to chorioretinal atrophy resulting from high myopia. In our patient, limited functional improvement was achieved due to chorioretinal atrophy in the macular region. Therefore, the fact that the severity of chorioretinal atrophy in the posterior pole will limit functional success should be considered prior to surgical intervention in these patients.

Switching to other anti-VEGF drugs is one option for overcoming tachyphylaxis in nAMD treatment. Bevacizumab and ranibizumab have similar protein composition and sites of action. Aflibercept is shown to be effective in patients with large PEDs that were insufficiently responsive to multiple bevacizumab and ranibizumab injections.^10^ Because of the higher binding affinity of aflibercept, we decided to switch ranibizumab to aflibercept and achieved a favorable anatomical outcome.

In conclusion, this case study revealed that different types of lesions can be seen not only in the course of nAMD but also at initial diagnosis. ICGA and OCT are the most important tools to diagnose coexisting lesions when suspected clinically.
